# Supplementary material for: Engineered Tn5 Variants Enhance CUT&Tag Fidelity for Transcription Factors
Source: bioRxiv. 2026 Oct 28:2026.10.27.762201. Preprint. [Version 1] doi: 10.64898/2026.10.27.762201 (PMC13733980; doi:10.64898/2026.10.27.762201)
Supplement: Supplementary file 1 [file NIHPP2026.10.27.762201V1-supplement-1.pdf]

bioRxiv preprint doi: https://doi.org/10.64898/2026.10.27.762201; this version posted October 28, 2026. The copyright holder for this preprint (which was not certified by peer review) is the author/funder, who has granted bioRxiv a license to display the preprint in perpetuity. It is made available under a CC-BY 4.0 International license.

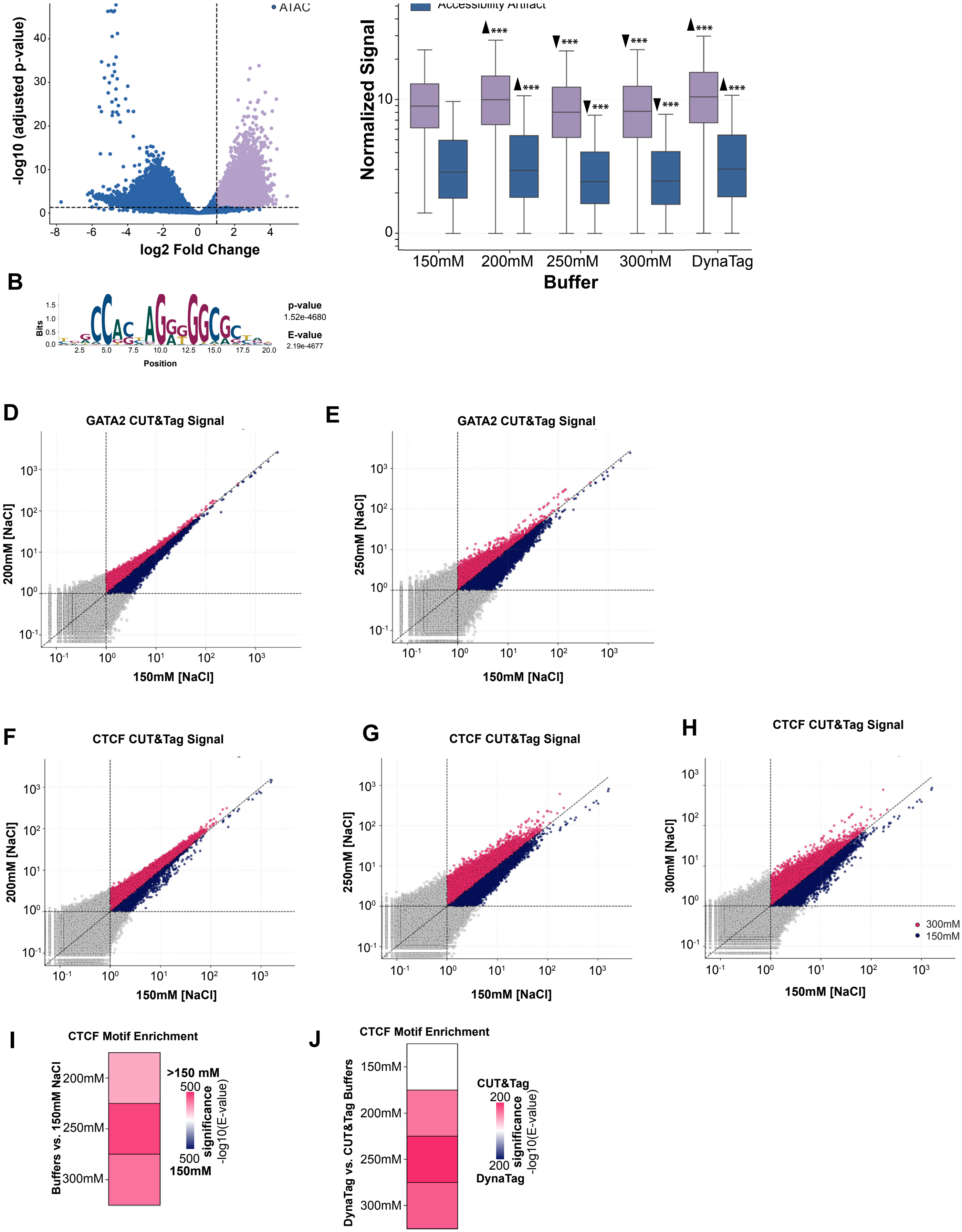

bioRxiv preprint doi: https://doi.org/10.64898/2026.10.27.762201; this version posted October 28, 2026. The copyright holder for this preprint (which was not certified by peer review) is the author/funder, who has granted bioRxiv a license to display the preprint in perpetuity. It is made available under a CC-BY 4.0 International license.

Supplementary Figure 1. Analysis of CTCF and GATA2 CUT&Tag signal recovery and specificity across ionic conditions. (A) Volcano plot of DESeq2 results comparing CTCF CUT&Tag generated at 150 mM NaCl with the curated K562 ATAC-seq reference. Peaks with log2 fold change > 1 and adjusted $P < 0.05$ were classified as CTCF-associated peaks and are shown in purple; remaining peaks comprise the operational accessibility-associated reference set and are shown in blue. **(B)** Sequence logo and enrichment statistics from AME comparing CTCF-associated peaks with the accessibility-associated reference set defined in (A). **(C)** Boxplot of normalized CTCF CUT&Tag signal across CTCF-associated and accessibility-associated peak sets defined in (A). Statistical comparisons are between the 150 mM NaCl condition and the 200, 250, and 300 mM NaCl and DynaTag conditions, with arrowheads indicating the direction of significant differences. **(D–E)** Scatterplots comparing normalized signal across a merged GATA2 peak set between 150 mM NaCl and 200 mM (D) or 250 mM (E) NaCl. Each point represents one peak; peaks with normalized signal ≥1 on both axes are colored according to the direction of the signal difference. **(F–H)** Same as (D–E), but comparing normalized signal across a merged CTCF peak set between 150 mM NaCl and 200 mM (F), 250 mM (G), or 300 mM (H) NaCl. **(I–J)** Heatmaps showing differential enrichment of the CTCF motif by AME, represented as −log10(E-value). In (I), pink indicates enrichment among peaks favored by NaCl concentrations >150 mM and navy indicates enrichment among peaks favored at 150 mM NaCl. In (J), pink indicates enrichment among peaks favored by the indicated NaCl condition and navy indicates enrichment among peaks favored by DynaTag.

bioRxiv preprint doi: https://doi.org/10.64898/2026.10.27.762201; this version posted October 28, 2026. The copyright holder for this preprint (which was not certified by peer review) is the author/funder, who has granted bioRxiv a license to display the preprint in perpetuity. It is made available under a CC-BY 4.0 International license.

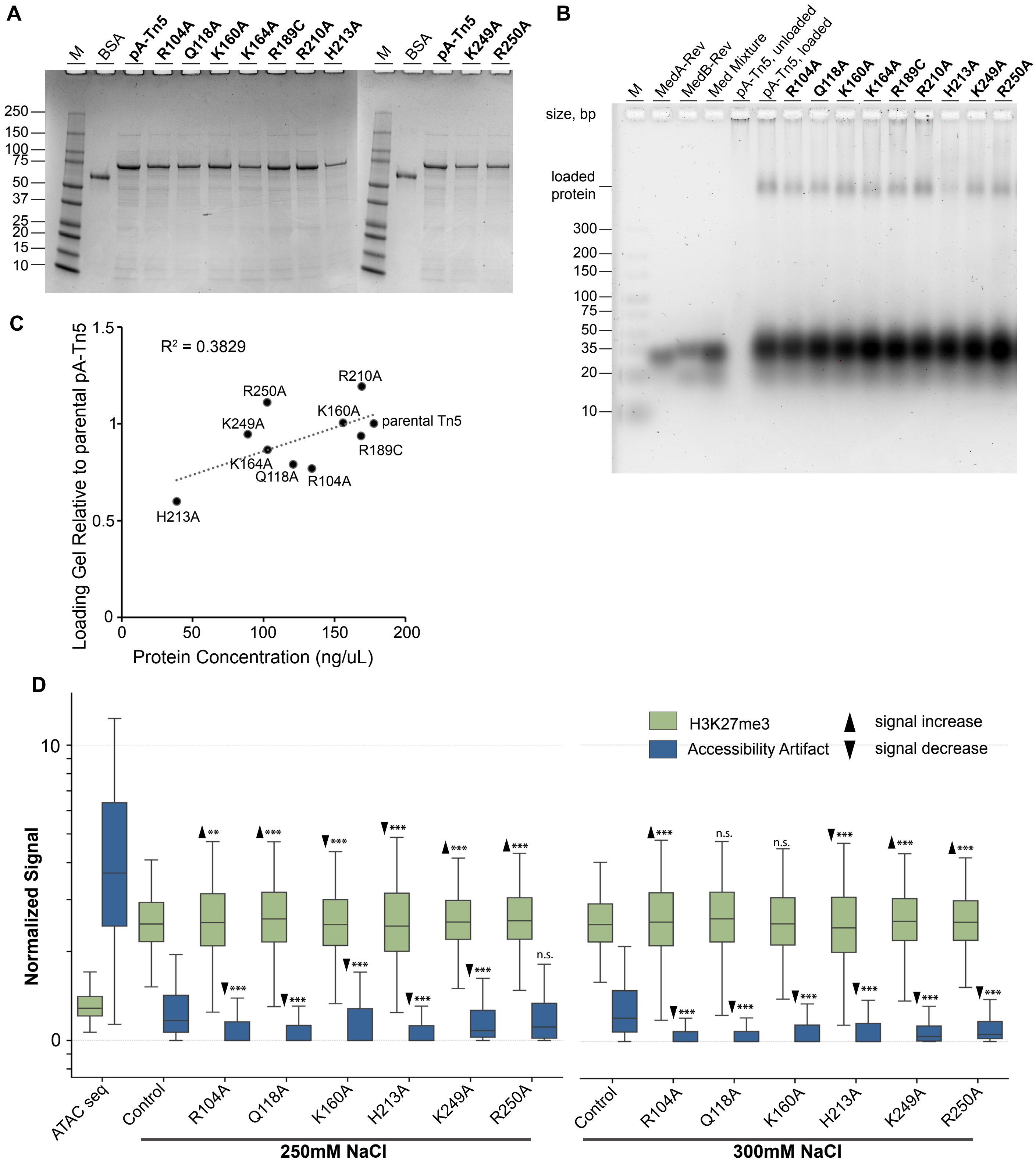

Supplementary Figure 2. Generation and functional characterization of mutant pA-Tn5 variants. (A) SDS-PAGE analysis of purified parental and mutant pA-Tn5 preparations used to quantify protein abundance prior to oligonucleotide loading. **(B)** Gel-based analysis of oligonucleotide loading for parental and mutant pA-Tn5 variants, with the shifted species representing assembled pA-Tn5 transposomes. **(C)** Quantification of oligonucleotide loading efficiency for each pA-Tn5 variant, calculated by normalizing the amount of shifted oligonucleotide in (B) to the corresponding amount of purified pA-Tn5 protein in (A). **(D)** Boxplots of normalized H3K27me3-associated and accessibility-associated signal for parental and mutant pA-Tn5 variants under 250 and 300 mM NaCl conditions. Statistical comparisons are between parental and mutant pA-Tn5 variants, with arrowheads indicating the direction of significant differences.

bioRxiv preprint doi: https://doi.org/10.64898/2026.10.27.762201; this version posted October 28, 2026. The copyright holder for this preprint (which was not certified by peer review) is the author/funder, who has granted bioRxiv a license to display the preprint in perpetuity. It is made available under a CC-BY 4.0 International license.

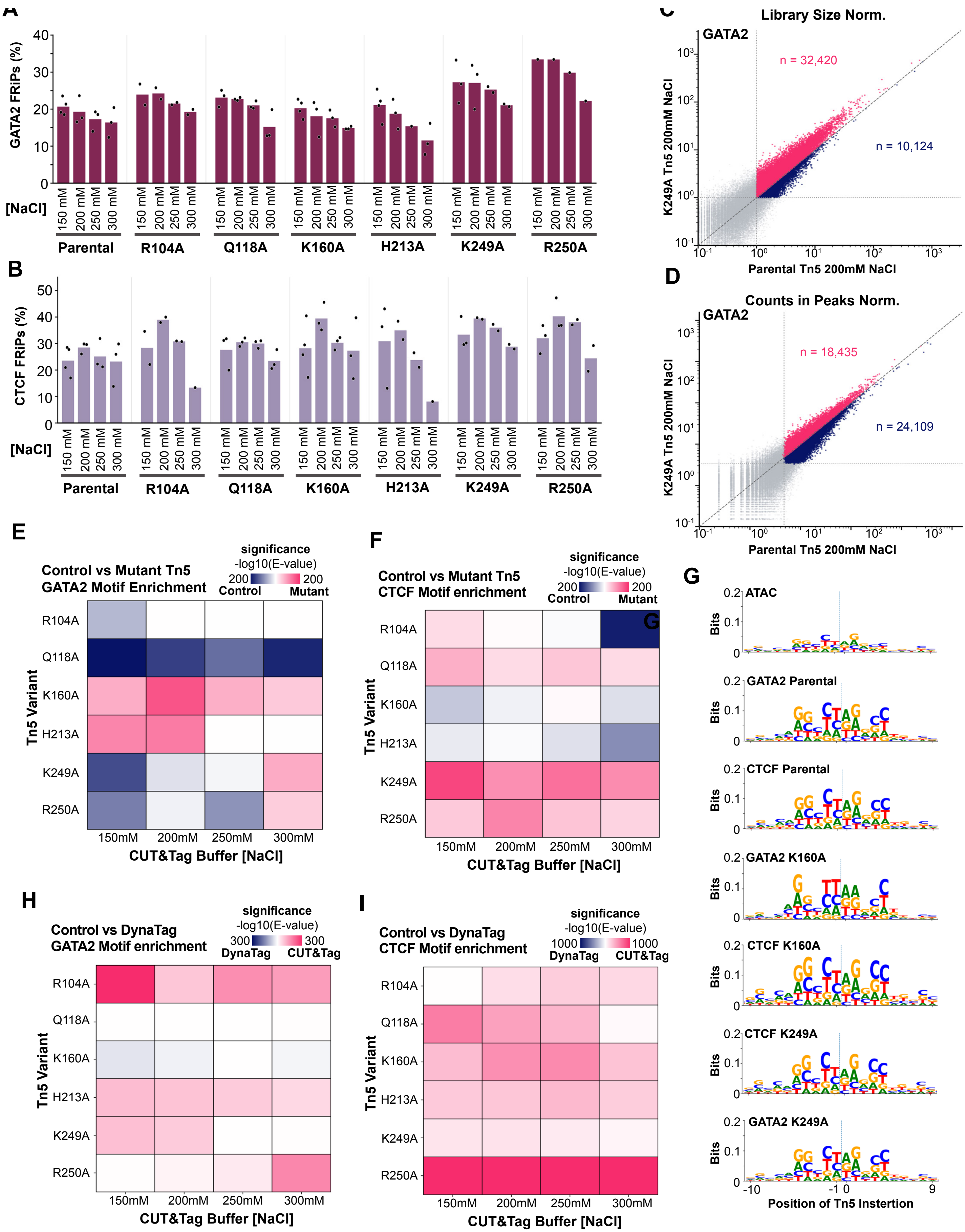

bioRxiv preprint doi: https://doi.org/10.64898/2026.10.27.762201; this version posted October 28, 2026. The copyright holder for this preprint (which was not certified by peer review) is the author/funder, who has granted bioRxiv a license to display the preprint in perpetuity. It is made available under a CC-BY 4.0 International license.

Supplementary Figure 3. Library normalization and Tn5 insertion preferences do not explain TF-dependent variant performance. (A,B) Fraction of reads in peaks (FRiP) for GATA2 (A) and CTCF (B) CUT&Tag libraries generated with parental pA-Tn5 or the indicated variants across 150, 200, 250, and 300 mM NaCl. Bars summarize replicate FRiP values; black dots indicate individual libraries. **(C,D)** Scatterplots comparing GATA2 CUT&Tag signal for K249A and parental pA-Tn5 at 200 mM NaCl after normalization to the total eligible library fragments (C; library-size normalization) or to the fragments falling within the consensus peak set (D; counts-in-peaks normalization). Each point represents one peak; pink indicates K249A-favored peaks and navy indicates parental-enzyme-favored peaks among those passing the signal threshold. **(E,F)** AME heatmaps showing differential enrichment of GATA2 (E) and CTCF (F) motifs between variant-favored and parental-enzyme-favored peak sets after normalization by fragments within the consensus peak set. Values are represented as signed −log10(E-value), with pink indicating enrichment in variant-favored peaks and blue indicating enrichment in parental-enzyme-favored peaks. The original whole-library-normalized signal threshold was retained to define peaks eligible for comparison. **(G)** Sequence logos showing Tn5 insertion-site sequence preferences in ATAC-seq and parental, K160A, and K249A CUT&Tag libraries for GATA2 and CTCF. Letter heights represent positional information in bits. **(H,I)** AME heatmaps comparing GATA2 (H) and CTCF (I) motif enrichment between NaCl-based CUT&Tag and DynaTag for the same indicated Tn5 variant. Pink indicates enrichment among NaCl-favored peaks and blue indicates enrichment among DynaTag-favored peaks, expressed as signed −log10(E-value).

bioRxiv preprint doi: https://doi.org/10.64898/2026.10.27.762201; this version posted October 28, 2026. The copyright holder for this preprint (which was not certified by peer review) is the author/funder, who has granted bioRxiv a license to display the preprint in perpetuity. It is made available under a CC-BY 4.0 International license.

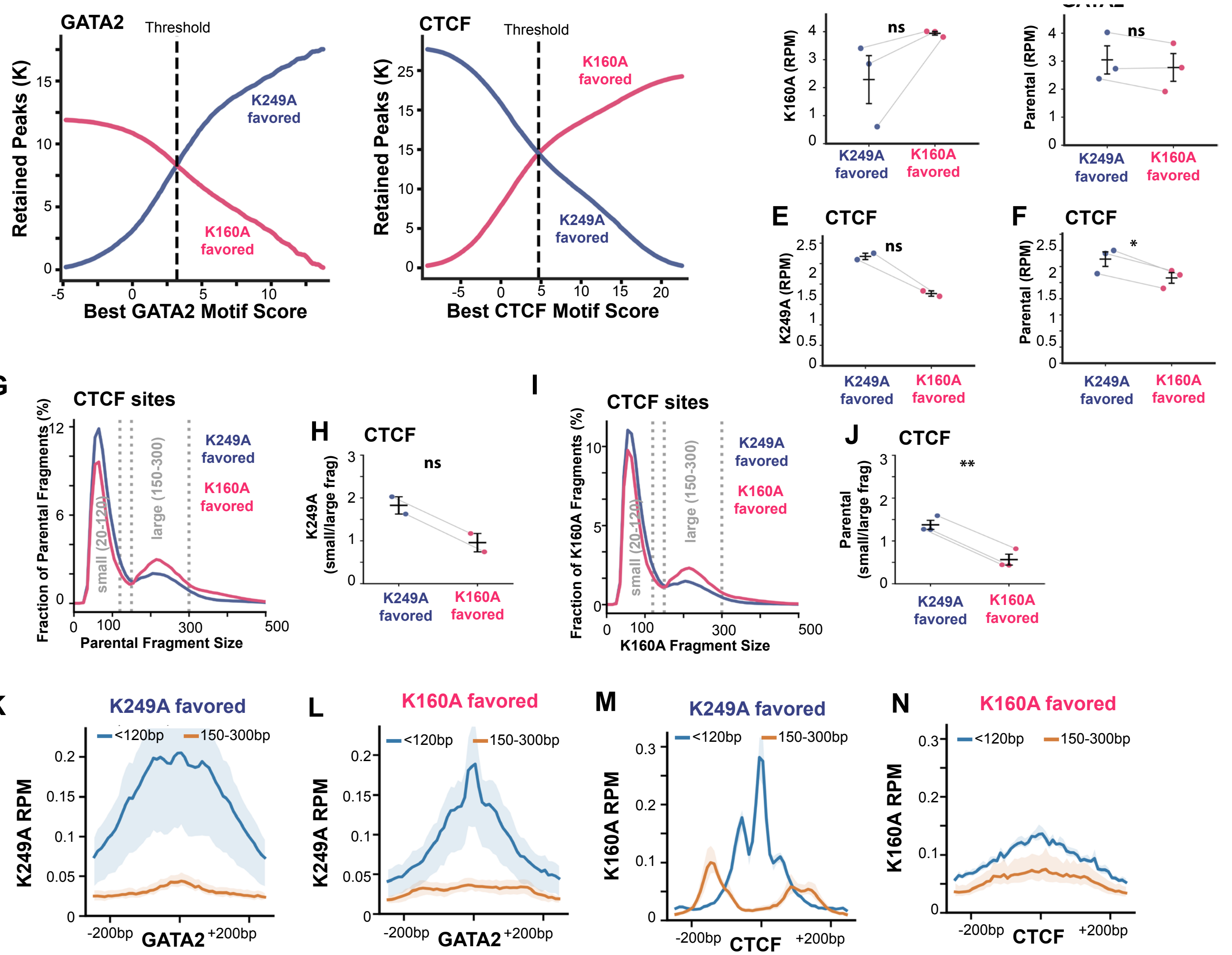

Supplementary Figure 4. Signal recovery and fragment organization at K160A- and K249A-preferred sites. (A,B) Numbers of GATA2 (A) and CTCF (B) sites retained in the opposing variant-preference/motif-score populations as the minimum motif-score threshold is varied. Dashed vertical lines indicate the thresholds used to define the selected populations in Fig. 4A,B. **(C,D)** Paired-library comparisons of mean normalized CUT&Tag signal (RPM per peak) across K249A- and K160A-preferred GATA2 sites for K160A (C) and parental pA-Tn5 (D). **(E,F)** Corresponding comparisons across K249A- and K160A-preferred CTCF sites for K249A (E) and parental pA-Tn5 (F). In (C–F), each point represents one library; lines connect measurements from the same library and black symbols indicate mean ± SEM. **(G,H)** Fragment-length distributions (G) and paired-library ratios of short to nucleosome-sized fragments (H) from parental pA-Tn5 CUT&Tag at the selected CTCF sites. **(I,J)** Corresponding fragment-length distributions (I) and short-to-nucleosome-sized fragment ratios (J) from K160A CUT&Tag at the CTCF sites. In (G,I), blue denotes K249A-preferred sites and pink denotes K160A-preferred sites; dashed lines delimit short (20–119 bp) and nucleosome-sized (150–299 bp) fragment ranges. **(K,L)** Motif-centered mean K249A CUT&Tag fragment-midpoint profiles for K249A-preferred (K) and K160A-preferred (L) GATA2 sites. **(M,N)** Corresponding mean K160A CUT&Tag profiles for K249A-preferred (M) and K160A-preferred (N) CTCF sites. In (K–N), blue denotes short fragments and orange denotes nucleosome-sized fragments; shaded areas indicate the range among individual libraries.

bioRxiv preprint doi: https://doi.org/10.64898/2026.10.27.762201; this version posted October 28, 2026. The copyright holder for this preprint (which was not certified by peer review) is the author/funder, who has granted bioRxiv a license to display the preprint in perpetuity. It is made available under a CC-BY 4.0 International license.

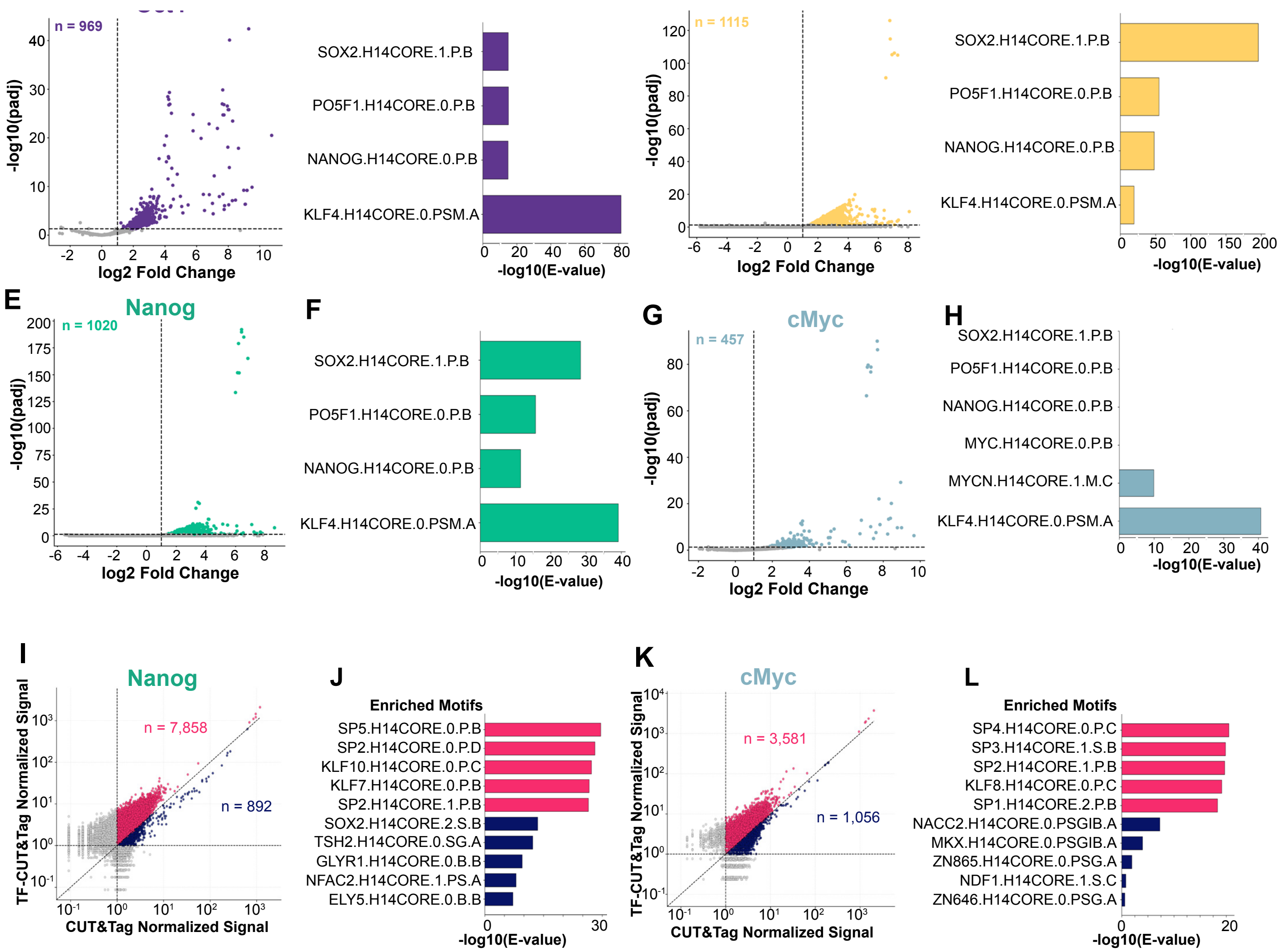

Supplementary Figure 5. Pluripotency-associated motif enrichment in TF-CUT&Tag varies among transcription factors. (A–H) DESeq2 and motif enrichment analyses comparing TF-CUT&Tag with H3K4me2 CUT&Tag for Oct4 (A,B), Sox2 (C,D), Nanog (E,F), and cMyc (G,H). Volcano plots (A,C,E,G) show differential signal between TF-CUT&Tag and H3K4me2 CUT&Tag. Bar plots (B,D,F,H) show AME enrichment of the indicated pluripotency-associated motifs, represented as −log10(E-value). **(I,K)** Scatterplots comparing normalized signal across merged peak sets between TF-CUT&Tag and standard CUT&Tag for Nanog (I) and cMyc (K). Each point represents one peak. Peaks with normalized signal ≥1 on both axes were classified as TF-CUT&Tag-favored if they fell above the diagonal (y = x) or standard-CUT&Tag-favored if they fell below it, and are highlighted in pink and blue, respectively. **(J,L)** Bar plots showing differential motif enrichment by AME between TF-CUT&Tag-favored and standard-CUT&Tag-favored peak sets for Nanog (J) and cMyc (L), represented as −log10(E-value). Pink indicates motifs enriched among TF-CUT&Tag-favored peaks and blue indicates motifs enriched among standard-CUT&Tag-favored peaks.
